# Should We Adopt Increased Dilutions for Indirect Immunofluorescence in Pediatric Anti-Centromere Antibody Testing? Insights from a Three-Year Retrospective Study †

**DOI:** 10.3390/children12010036

**Published:** 2024-12-28

**Authors:** Mehmet Soylu, Raziye Burcu Taşkın, Gülçin Aytaç, Güzide Aksu, Seyfi Durmaz, Miray Karakoyun, Şaziye Rüçhan Sertöz

**Affiliations:** 1 Department of Medical Microbiology, Faculty of Medicine, Ege University, Izmir 35100, Turkey; mehmet.soylu@ege.edu.tr (M.S.); ruchan.sertoz@ege.edu.tr (Ş.R.S.); 2 Department of Pediatric Rheumatology, Faculty of Medicine, Ege University, Izmir 35100, Turkey; raziyeburcu.taskin@saglik.gov.tr (R.B.T.); gulcin.aytac@saglik.gov.tr (G.A.); guzide.aksu@ege.edu.tr (G.A.); 3 Department of Public Health, Faculty of Medicine, Ege University, Izmir 35100, Turkey; 4 Department of Pediatric Gastroenterology, Faculty of Medicine, Ege University, Izmir 35100, Turkey; miray.karakoyun@ege.edu.tr

**Keywords:** centromere protein B, centromere, autoimmunity, fluorescent antibody technique, immunoblotting

## Abstract

Background/Objectives: Systemic autoimmune rheumatic diseases (SARDs) pose diagnostic challenges, particularly in pediatric populations, due to their diverse presentations and overlapping symptoms. This study aimed to evaluate the diagnostic concordance between indirect immunofluorescence (IIF) at different dilution levels (1/80 and 1/640) and immunoblot findings for anti-centromere antibody (ACA) positivity. Additionally, the clinical significance of ACA positivity and its association with SARDs in pediatric patients was assessed. Methods: This retrospective, cross-sectional study included 58 pediatric patients evaluated for anti-nuclear antibody (ANA) testing at Ege University Hospital from 2019 to 2021. IIF was performed using HEp-20-10 cells and immunoblot testing was conducted to assess CENP-B reactivity. Statistical analyses included chi-square tests, correspondence analysis, and regression modeling to explore the relationship between IIF titers, immunoblot findings, and SARD diagnoses. Results: Among the patients, 62.1% were diagnosed with SARD. Higher IIF titers (≥1/640) were strongly associated with CENP-B 3+ immunoblot positivity, while lower titers (1/80 and 1/320) correlated with CENP-B 1+. Patients with IIF positivity at 1/80 were 15.89 times more likely to have SARD (*p* < 0.001). Correspondence analysis revealed significant associations between IIF dilution levels and immunoblot reactivity (χ^2^ = 37.574, *p* < 0.000). Gender and age were not significant predictors of SARD positivity. Conclusions: This study highlights the diagnostic value of higher IIF dilution levels (≥1/640) in improving ACA detection and SARD diagnosis in pediatric patients. Incorporating complementary diagnostic tools, such as immunoblot testing, can enhance diagnostic accuracy. These findings support adopting higher IIF cutoff levels in clinical practice for pediatric populations.

## 1. Introduction

Systemic autoimmune rheumatic diseases (SARDs) are complex disorders that have long been a subject of interest and pose a diagnostic challenge because of their diverse clinical presentations and overlapping symptoms [1]. Anti-nuclear antibodies (ANA) have been used as valuable serological markers for diagnosing autoimmune diseases. Indirect immunofluorescence microscopy (IIF) using Hep-2 cells is considered the gold standard method for ANA detection and allows the detection of antibody reactivity against nuclear and/or nonnuclear components [2].

While ANA positivity for autoimmune disease detection in adults can vary from 1/40 to 1/160 or above, in pediatric cases, dilutions exceeding 1/640 are recommended to minimize false positives and maintain better diagnostic accuracy [3,4,5].

The increased threshold reflects the difficulties associated with interpreting ANA patterns in children, as low-titer positivity may be transient or nonspecific. Despite its widespread use, the IIF method has limitations. These include sensitivity and specificity issues, dependence on subjective interpretation, and the need for highly trained personnel, which pose potential pitfalls. Furthermore, the detection of multiple autoantibodies corresponding to a single pattern and the low expression of SS-A/Ro in Hep-2 cells necessitate the use of complementary diagnostic tools [5,6].

Anti-centromere antibodies (ACA) are a unique class of autoantibodies that target centromere proteins, which are structural components of chromosomes responsible for proper cell division. ACA was first identified in the 1980s and has since emerged as a valuable marker for CREST syndrome (calcinosis, Raynaud’s phenomenon, esophageal dysmotility, sclerodactyly, and telangiectasia) in adults [7]. Over four decades of research have revealed a diverse array of ACA targets, including anti-centromere proteins (CENPs) CENP-A, CENP-B, CENP-C, and CENP-F, with CENP-B autoantibodies playing prominent roles [8,9].

IIF is a key method for detecting ACA, particularly the AC-3 IIF pattern, which is characterized by discrete coarse speckles scattered across interphase nuclei and aligned with the chromatin mass in mitotic cells. This pattern is strongly associated with anti-CENP-A and anti-CENP-B antibodies, which are detected in various disease-specific profiles [10,11]. ACA positivity has been observed in limited cutaneous systemic sclerosis (SSc) and is predictive of its onset when associated with Raynaud’s phenomenon [12]. In primary biliary cholangitis (PBC), ACA positivity is reported in a subset of patients, often overlapping with systemic sclerosis [13]. ACA-positive Sjögren’s syndrome (SS) represents a distinct clinical subset characterized by systemic sclerosis-like features such as Raynaud’s phenomenon and sclerodactyly alongside severe exocrine gland dysfunction, while displaying a lower prevalence of hematologic abnormalities like leukocytopenia and thrombocytopenia [14]. Furthermore, ACA positivity has been observed in systemic lupus erythematosus (SLE), particularly in cases with overlapping SSc features [15].

A comprehensive evaluation of ACA positivity is essential because of the complex interplay between clinical presentation, disease course, and ACA positivity, particularly in the pediatric population.

The primary aim of this study was to evaluate the diagnostic concordance between IIF at dilutions of 1/80 and 1/640 and a line immunoassay (LIA) for detecting anti-centromere antibody (ACA) positivity in pediatric patients. Additionally, this study intended to assess the clinical relevance of ACA positivity by investigating its association with systemic autoimmune rheumatic diseases (SARDs) in this population over a three-year period at a university hospital.

## 2. Materials and Methods

This study was conducted on pediatric patient samples accepted to the Medical Microbiology Laboratory of Ege University Hospital for ANA screening between 2019 and 2021. The study employed a cross-sectional and methodological analysis to assess the relationship between AC-3 IIF titers and immunoblot findings. Additionally, the study aimed to determine the significance of gender, age, immunoblot results, and AC-3 IIF titers in clinical diagnosis.

### 2.1. Participants

Inclusion criteria were pediatric patients who provided samples between 2019 and 2021 for ANA testing and had CENP-B immunoblot positivity. Patients with negative CENP-B results or duplicate records were excluded. A total of 58 pediatric patients met the inclusion criteria. Patient demographics, including age and gender, as well as clinical data, were retrospectively obtained from hospital records.

### 2.2. Data Collection

IIF ANA testing was performed utilizing HEp-20-10 and monkey liver cell substrates manufactured by Euroimmun AG (Lübeck, Germany). The initial screening dilution was established at 1:80, with positive specimens subsequently subjected to serial dilutions of 1:320, 1:640, 1:1280, 1:2560, and 1:5120, contingent upon the immunofluorescence intensity observed in the preliminary screening. Samples demonstrating persistently high immunofluorescence signals underwent progressive dilution until reaching the 1:5120 threshold.

The ANA IIF patterns were evaluated according to the International Consensus on ANA Patterns (ICAP) guidelines. IIF patterns which were characterized by discrete coarse speckles scattered across interphase nuclei and aligned with the chromatin mass in mitotic cells were diagnosed as AC-3 [11]. The immunoblot (LIA) was conducted using the EUROLINE ANA Profile 3 plus DFS70 (IgG) test kit, and all procedures were automated using the EUROBlotOne system. The bands were analyzed using EUROLine Scan software 3.4.

The study data, including laboratory results and demographic information, were retrospectively collected through the hospital’s information system. The validity and reliability of the data were ensured by using standardized test kits and automated systems in line with international clinical standards.

### 2.3. Variables and Measurements

The dependent variable in this study was the clinical diagnosis, which was categorized into two groups: patients diagnosed with SARDs and those without SARDs (non-SARD). The diagnosis was based on clinical evaluation and laboratory test results, including both IIF and immunoblot findings. The independent variables included gender (male and female), age (as a continuous variable), immunoblot results (CENP-B 1+, CENP-B 2+, CENP-B 3+), and IIF titers at various dilution levels (1/80, 1/320, 1/640, etc.). These factors were analyzed for their potential influence on clinical diagnosis.

### 2.4. Statistical Analysis

Data were analyzed using SPSS Statistics software version 25.0 (IBM Corp., Armonk, NY, USA). Descriptive statistics (mean, percentage) were used to summarize the variables. The chi-square test was applied to compare categorical variables. Correspondence analysis was conducted to explore the relationship between IIF dilution levels and immunoblot reactivity. Additionally, regression analysis was performed to assess the association between gender, age, immunoblot results, IIF titers, and the likelihood of a positive SARD diagnosis. Statistical significance was set at *p* < 0.05.

### 2.5. Strengths and Limitations

The study’s strengths include the use of pediatric samples and the comparison of two distinct laboratory methods. However, limitations include the retrospective design, which limits the establishment of causal relationships, and the data being sourced from a single center, which may restrict the generalizability of the findings. Furthermore, uncertainties such as the significance of the CENP-B 3+ association with a wide confidence interval (e.g., 95% CI: 1.00–106.73) and wide confidence intervals in IIF titers (e.g., 95% CI: 1/80: 3.71–68.13) can be resolved with larger sample sizes. 

### 2.6. Ethical Considerations

This study was approved by the Ethics Committee of Ege University Medical Research (Approval No: 23-9.1T/12, Date: 21 September 2023). Since the study was retrospective, patient data were anonymized, and no additional informed consent was required. All data were handled following ethical standards, ensuring confidentiality. There were no conflicts of interest or financial support for this study.

## 3. Results

Among the patients, 62.1% were diagnosed with SARDs, while 37.9% were classified as non-SARD. The gender distribution shows a predominance of female patients (72.4%), with males accounting for 27.6%. The mean age of the patients was 11.7 years (SD = 4.1). The descriptive characteristics of pediatric patients with CENP-B positivity detected via immunoblot are presented in the Supplementary Table S1.

In terms of immunoblot results, CENP-B 1+ was the most frequently detected result (39.7%), followed by CENP-B 2+ at 36.2% and CENP-B 3+ at 24.1%. Regarding AC-3 IIF titers, 27.6% of patients were negative, while a range of positive titers was observed: 6.9% had a titer of 1/80, 19% at 1/320, 19% at 1/640, 13.8% at 1/1280, 8.6% at 1/2560, and 5.2% at 1/5120.

Regarding the distribution of positivity levels in the SARD group, systemic sclerosis showed a mix of 2+ (●) and 3+ (■) positivity with higher dilutions (1/2560 and 1/5120). Sjögren’s syndrome presented mostly with 1+ (▲) and 2+ (●) positivity, appearing at the 1/320 and 1/640 dilutions. Familial Mediterranean fever had the most widespread positivity, with 1+, 2+, and 3+ positivity across a range of dilutions from 1/80 to 1/2560. Juvenile idiopathic arthritis also showed positivity across multiple dilutions but appeared more often at the 1+ (▲) and 2+ (●) levels. In the non-SARD group, arthralgia (nonrheumatic) displayed high 1+ (▲) and 2+ (●) positivity, and other non-SARD diseases (e.g., nephrotic syndrome and autoimmune hepatitis) tended to show lower positivity (mostly 1+ or 2+), with fewer occurrences of 3+ positivity (Table 1).

In the study group, thirteen patients had other positive immunoblot bands. The most common bands were DFS70 (61.5%), Ro-52 (23%), dsDNA (7.6%), and SS-B (7.6%). Additionally, 37 out of 58 patients had another IIF-pattern positivity. These patterns were distributed as AC-4,5 nuclear speckled (64.8%), AC-1 nuclear homogeneous (21.6%), AC-2 nuclear dense fine speckled (10.8%), AC-10 punctate nucleolar (2.6%), and AC-6 multiple nuclear dots (2.6%). The largest group of secondary pattern-positive IIF patients were patients with the AC-4-5 nuclear speckled pattern, and 29.1% of this group was IIF AC-3 centromere pattern-negative. Additionally, 75% of this group had a history of SARDs. These data are presented in Supplementary Table S2.

When categorized by specific cut-off points for IIF positivity, 72.4% of the patients were positive at a titer of 1/80, while 46.6% were positive at a titer of 1/640.

The correspondence analysis revealed a significant association between the two variables (χ^2^ = 37.574, *p* < 0.000). The first dimension accounted for 83.2% of the variance, indicating that it captured most of the variability in the relationship between dilution levels and immunoblot outcomes.

Higher dilution levels, such as 1/640 and 1/1280, were strongly associated with CENP-B 3+ results, reflecting stronger immune responses. In contrast, lower dilution levels and negative results were more closely aligned with CENP-B 1+, indicating weaker reactivity. The second dimension explained an additional 16.8% of the variance, providing supplementary differentiation between the categories.

**Figure 1:**
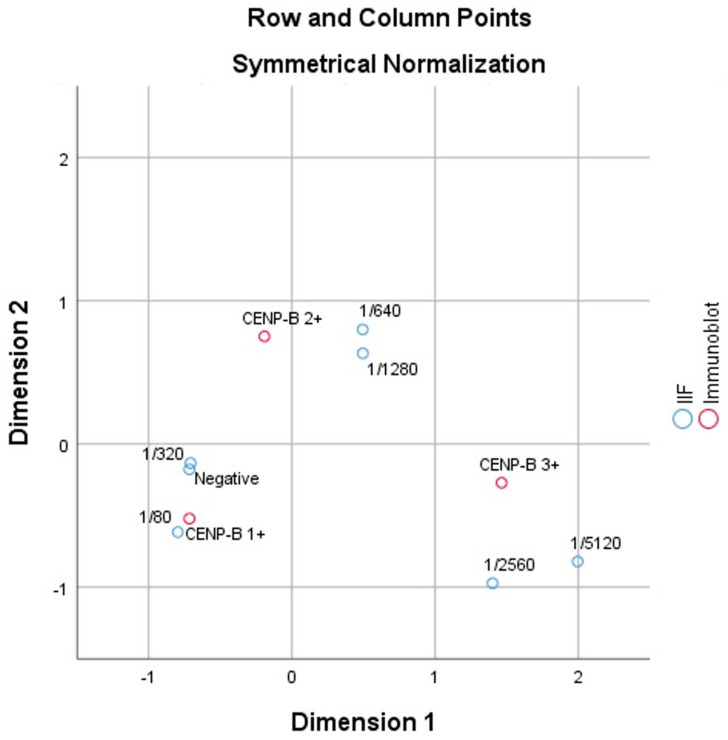
The two-dimensional plot (row and column points) visualizes the relationships between IIF dilution levels and immunoblot results.

These findings align with the existing literature, where higher dilution levels are typically associated with stronger immunological reactions. This study confirms the utility of correspondence analysis in identifying and visualizing the relationships between diagnostic categories in immunological testing (Figure 1).

### 3.1. SARD

A correspondence analysis was conducted to investigate the relationship between IIF dilution levels and immunoblot reactivity within the SARD group dataset. The analysis revealed a statistically significant association between the two variables (χ^2^ = 31.214, *p* = 0.002), indicating that dilution levels and immunoblot results were not independent. The analysis identified two dimensions that captured the variance in the data: Dimension 1 accounted for 74.1% of the total variance, representing the primary relationship between higher IIF dilution levels and stronger immunoblot reactivity (CENP-B 3+). Dimension 2 explained an additional 25.9% of the variance, providing further differentiation between the immunoblot categories.

The results showed the following clear pattern: Higher dilution levels (greater than 1/640) were strongly associated with CENP-B 3+, suggesting stronger immune reactivity. Lower dilution levels (e.g., negative, 1/80, and 1/320) were more frequently associated with CENP-B 1+, indicating weaker immunoblot responses (Table 2).

### 3.2. Non-SARD

The correspondence analysis conducted on the filtered non-SARD group dataset revealed a not statistically significant relationship between IIF dilution levels and immunoblot results (χ^2^ = 17.469, *p* = 0.133). Most of the variance (67.8%) was explained by Dimension 1, indicating that the most important distinction lies between higher and lower dilution levels. Dimension 2 accounted for an additional 32.2% of the variance, providing further differentiation between the categories.

Higher dilution levels (such as 1/1280) were strongly associated with CENP-B 3+ results, reflecting stronger immune reactivity. Negative results were linked to CENP-B 1+, indicating weaker reactivity. Negative, 1/320, and 1/640 showed association with CENP-B 2+ (Table 2).

The study analyzed the relationship between gender, age, immunoblot test results, AC-3 IIF titers, and the likelihood of SARD positivity. The findings indicate that neither gender nor age had a significant effect on SARD positivity. In the immunoblot test, CENP-B 3+ was associated with an increased likelihood of SARD positivity (OR = 11.92), and this relationship was statistically significant (*p* = 0.027). On the other hand, CENP-B 2+ did not demonstrate a significant association with SARD positivity (OR = 1.01, *p* = 0.989).

The strongest associations were observed with AC-3 IIF titers. Patients with a positive AC-3 IIF (1/80) titer were found to have a 15.89 times higher likelihood of SARD positivity compared to those with negative titers, and this relationship was highly significant (*p* < 0.001). While a positive AC-3 IIF (1/640) titer also showed a significant association, its effect was not as strong as that of the IIF (1/80) titer. These findings suggest that AC-3 IIF titer positivity, particularly at the 1/80 dilution, is an important serological indicator for SARD diagnosis. The Nagelkerke R-square values indicated that IIF (1/80) titer positivity was the most explanatory variable for SARD positivity (R^2^ = 0.361) (Table 3).

## 4. Discussion

In this study, the use of a 1/640 or higher dilution was consistently associated with stronger CENP-B immunoblot positivity (2+ and 3+) and a closer relationship to SARDs. These findings are supported by our analyses presented in Figure 1 and Table 3. Compared with the cutoff value of 1/80, the higher threshold minimizes false positives, which are often encountered in pediatric ANA/ACA interpretation [4]. In this group, more positive clusters (+2 and +3) were consistently identified at ≥1/640, further supporting the validity of this cutoff point, and the presence of SARDs significantly influenced the pattern of ACA positivity. This finding aligns with previous studies that recommended higher IIF dilution thresholds in pediatric populations to improve diagnostic accuracy and reduce false-positive results [16].

The observed high concordance between the IIF (1/640 cutoff) and LIA results strengthens the argument for using an LIA as a complementary tool. LIAs offer advantages such as increased specificity for CENP-B, a key ACA target. This can be particularly beneficial in confirming ACA positivity detected by IIF, especially in patients with borderline titers or overlapping ANA patterns. Integrating an LIA into the diagnostic workup can increase diagnostic confidence and streamline patient management [17].

The sex distribution within the study population did not significantly alter the concordance between the IIF and immunoblot methods. However, a higher prevalence of female patients aligns with the consensus that SARDs tend to occur more frequently in females [18]. The predominance of certain immunoblot bands, such as DFS70, Ro-52, and others, coupled with secondary IIF patterns indicates broader autoantibody profiles in pediatric patients, which may aid clinicians in determining a more comprehensive diagnosis and understanding potential disease overlap [19,20].

In children without a history of SARDs, low positive immunoblot results (+1) were associated mainly with IIF-negative cases. This observation suggests that low-level LIA positivity might not always translate to clinically relevant ACA presence, particularly when it is not accompanied by a positive IIF pattern. This finding highlights the need for the cautious interpretation of such results and underscores the importance of combining LIAs with IIF for a more comprehensive evaluation, especially in children without established SARDs [21]. In our study, three cases demonstrated CENP-B positivity (CENP-B 2+) without the AC-3 IIF pattern, instead showing the following alternative IIF patterns: AC-4/5 at dilutions of 1/640 and 1/5120, and AC-2 at 1/320. This raises the possibility of cross-reactivity between centromere-associated antigens and nucleolar or homogeneous patterns. However, as highlighted in the literature, antibody cross-reactivity does not necessarily imply pathogenic significance or a direct involvement in disease processes [22,23].

The absence of AC-3 IIF in these cases may reflect heterogeneity in immune responses or overlaps in antigen reactivity. Similar findings have been noted in other studies, where cross-reactivity is often attributed to shared sequences or structures without clear functional consequences. While our data suggest a potential cross-reactive mechanism, further studies are needed to clarify its clinical relevance.

In our study, eleven patients were diagnosed with familial Mediterranean fever (FMF) according to their medical records. While potentially incidental, this observation warrants further investigation. FMF is an autoinflammatory disorder caused by mutations in the MEFV gene, which encodes the protein pyrin. Pyrin plays a critical role in regulating the inflammatory response through inflammasome assembly. This activation can potentially lead to the production of autoantibodies such as ACAs, although the exact mechanisms remain unclear [24]. Tanaka et al. reported the coexistence of ACA positivity and FMF, with elevated levels of IL-18 observed; however, our study did not investigate specific cytokine profiles, such as IL-18 [25]. Future research, similar to the case studies mentioned above, could explore the role of specific inflammatory mediators and their potential contribution to ACA production in FMF patients. FMF is primarily characterized by recurrent febrile episodes and serositis, and, while it is traditionally classified as an autoinflammatory disease, there is emerging evidence suggesting that patients with FMF may also exhibit autoimmune features, including the presence of various autoantibodies. A case report highlighted the progression of an FMF phenotype into rheumatoid arthritis (RA), indicating a potential overlap between autoinflammatory and autoimmune mechanisms [26].

A major limitation of this study is the small sample size. This makes it difficult to generalize the findings to the entire pediatric population and reduces the power of statistical analyses. Additionally, celiac disease was evaluated within the SARD group. Several studies have highlighted celiac disease and rheumatic diseases as disorders arising from similar mechanisms through different antigens [27]. As an additional limitation, our study did not include a detailed subgroup analysis of ANA staining patterns across different clinical conditions, which could have further elucidated the relationship between specific patterns and disease subtypes.

In conclusion, our findings underscore the complex nature of interpreting autoimmune markers in pediatric populations, demanding a detailed approach in clinical practice. The statistical analysis highlights the importance of using the higher IIF cutoff value of ≥1/640 in pediatric patients. This threshold enhances the specificity of ACA detection and is strongly associated with higher immunoblot positivity grades, thereby improving diagnostic precision and assisting in the accurate identification of children at risk for SARDs. The coexistence of autoimmune markers with various clinical conditions, including FMF, underscores the importance of a multidisciplinary approach to diagnosis and management in pediatric rheumatology.

Future studies with larger study groups should delve into the specificities of autoantibodies associated with ACA pattern positivity and their clinical implications, providing a more refined understanding of autoimmune processes in pediatric patients.

## Figures and Tables

**Table 1:** Distribution of indirect immunofluorescence microscopy titers among patients with and without SARDs, with symbols indicating immunoblot positivities.

		Indirect Immunflorescence Microscopy						
		Negative	1/80	1/320	1/640	1/1280	1/2560	1/5120
SARD	Systemic sclerosis	●		▲	■		■■	■
	Sjögren’s syndrome			▲	■	■		
	Behçet’s disease	●						■
	Celiac disease			●	▲●■			■
	Undifferentiated connective tissue disease			▲				
	Juvenile dermatomyositis					●		
	Juvenile idiopathic arthritis	▲		▲	●●	●●	■	
	* Familial Mediterranean fever		▲▲▲	▲▲●	●■	▲■	■	
NON-SARD	Arthralgia depending on nonrheumatic disease	▲▲▲▲▲ ▲▲▲●●●●	●	▲●	●●		▲	
	Nephrotic syndrome			●				
	Autoimmune hepatitis					●■		
	Primary biliary cirrhosis	▲						

Immunoblot CENP-B positivity (each symbol indicates a patient). ▲: +1 positive, ●: +2 positive, ■: +3 positive. * Familial Mediterranean fever included in SARD group because of rheumatic nature of disease.

**Table 2:** Summary of correspondence analysis for IIF dilution levels and immunoblot classifications.

IIF	Immunoblot									
	CENP-B 1+			CENP-B 2+			CENP-B 3+			Total
	Total	SARD	Non SARD	Total	SARD	Non SARD	Total	SARD	Non SARD	
Negative	10	1	9	6	2	4	0	0	0	16
1/80	3	3	0	1	0	1	0	0	0	4
1/320	7	6	1	4	2	2	0	0	0	11
1/640	1	1	0	6	4	2	4	4	0	11
1/1280	1	1	0	4	3	1	3	2	1	8
1/2560	1	0	1	0	0	0	4	4	0	5
1/5120	0	0	0	0	0	0	3	3	0	3
Total	23	12	11	21	11	10	14	13	1	58

**Table 3:** Predictors of SARD positivity.

		n	%		95% C.I. for OR		*p*	Nagelkerke R Square
				Odds Ratio	Lower	Upper		
Gender	Male (ref)	10	62.5					
	Female	26	61.9	1.03	0.31	3.37	0.967	<0.001
Age (mean ± sd)	(11.7 ± 4.1)			0.90	0.80	1.10	0.272	0.029
Immunoblot	CENP-B 1+ (ref)	12	52.2%					
	CENP-B 2+	11	52.4%	1.01	0.31	3.30	0.989	0.193
	CENP-B 3+	13	92.9%	11.92	1.33	106.73	0.027	0.193
IIF (1/80)	Negative (ref)	3	18.8					
	Positive	33	78.6	15.89	3.71	68.13	<0.001	0.361
IIF (1/640)	Negative (ref)	15	48.4					
	Positive	21	77.8	3.73	1.18	11.77	0.025	0.122

## Data Availability

The data presented in this study are available at reasonable request from the corresponding author. The data are not publicly available due to ethical reasons.

## Supplementary Materials

The following supporting information can be downloaded at: https://www.mdpi.com/article/10.3390/children12010036/s1, Table S1: Descriptive Characteristics of Pediatric Patients with CENP-B Positivity Detected via Immunoblot; Table S2: Dataset table including diagnosis of patients.
