# Supplementary material for: Should We Adopt Increased Dilutions for Indirect Immunofluorescence in Pediatric Anti-Centromere Antibody Testing? Insights from a Three-Year Retrospective Study
Source: Children (Basel). 2024 Dec 28;12(1):36. doi: 10.3390/children12010036 (PMC11763642; doi:10.3390/children12010036)
Supplement: Supplementary file 1 [file children-12-00036-s001.zip › children-3351577-supplementary.pdf]

SUPPLEMENTARY MATERIALS

Supplementary Table S1. Descriptive Characteristics of Pediatric Patients with CENP-B Positivity Detected via Immunoblot

	n	%
**Gender**		
Female	42	72.4
Male	16	27.6
**Years (mean & sd)**	11.7	4.1
**Immunblot**		
Centromere 1+	23	39.7
Centromere 2+	21	36.2
Centromere 3+	14	24.1
**IIF**		
Negative	16	27.6
1/80	4	6.9
1/320	11	19.0
1/640	11	19.0
1/1280	8	13.8
1/2560	5	8.6
1/5120	3	5.2
**IIF cut off value(1/80)**		
Negative	16	27.6
Positive	42	72.4
**IIF (1/640)**		
Negative	31	53.4
Positive	27	46.6
**Diagnosis**		
nonSARD	22	37.9
SARD	36	62.1

Supplementary Table S2. Dataset table including diagnosis of patients

PATIENT NUMBER	CENTROMERE PATTERN POSITIVITY IIF DILUTION	SECONDARY IIF PATTERN	AGE	GENDER	CENP-B POSITIVITY	IMMUNOBLOT SECONDARY PATTERN	DIAGNOSIS
1	1/80	1/320 AC-4,5	13	Male	1+	SS-B 2+	FAMILIAL MEDITERRANEAN FEVER
2	1/80	1/320 AC-4,5	18	Female	1+		FAMILIAL MEDITERRANEAN FEVER
3	1/80	1/640 AC-4,5	4	Female	1+		FAMILIAL MEDITERRANEAN FEVER
4	1/80	1/640 AC-4,5	5	Female	2+		NON-RHEUMATIC ARTHRALGIA
5	1/320	1/320 AC-4,5	6	Female	1+		FAMILIAL MEDITERRANEAN FEVER
6	1/320		14	Female	1+	DFS 1+	UNDIFF. CONNECTIVE TISSUE DISEASE
7	1/320		12	Female	1+	DFS 1+	SJOGREN
8	1/320	1/320 AC-2	16	Male	1+	DFS 3+	NON-RHEUMATIC ARTHRALGIA
9	1/320	1/320 AC-2	16	Male	1+	DFS 3+	JUVENIL IDIOPATHIC ARTHRITIS
10	1/320	1/320 AC-2	7	Male	2+	DFS 3+	FAMILIAL MEDITERRANEAN FEVER
11	1/320		12	Female	2+		CELIAC DISEASE
12	1/320		18	Male	1+		FAMILIAL MEDITERRANEAN FEVER
13	1/320		11	Male	1+		SYSTEMIC SCLEROSIS
14	1/320		7	Female	2+		NEPROTIC SYNDROME
15	1/320		14	Male	2+		NON-RHEUMATIC ARTHRALGIA
16	1/640	1/160 AC-4,5	5	Male	3+		FAMILIAL MEDITERRANEAN FEVER
17	1/640	1/320 AC-4,5	8	Male	2+		JUVENIL IDIOPATHIC ARTHRITIS
18	1/640	1/640 AC-4,5	13	Female	2+		CELIAC DISEASE
19	1/640	1/5120 AC-4,5	13	Female	2+	Ro-52 3+	JUVENIL IDIOPATHIC ARTHRITIS
20	1/640		9	Male	1+		CELIAC DISEASE
21	1/640	1/160 AC-1	7	Female	2+		NON-RHEUMATIC ARTHRALGIA
22	1/640	1/640 AC-1	14	Female	2+		FAMILIAL MEDITERRANEAN FEVER
23	1/640		15	Female	2+		NON-RHEUMATIC ARTHRALGIA
24	1/640		7	Female	3+		CELIAC DISEASE
25	1/640		6	Female	3+		SJOGREN
26	1/640	1/640 AC-10	5	Female	3+		SYSTEMIC SCLEROSIS

27	1/1280	1/320 AC-4,5	16	Female	1+		FAMILIAL MEDITERRANEAN FEVER
28	1/1280	1/640 AC-4,5	12	Female	3+		SJOGREN
29	1/1280	1/1280 AC-4,5	10	Female	2+		JUVENIL DERMATOMYOSITIS
30	1/1280	1/5120 AC-4,5	13	Female	2+	DFS 3+	JUVENIL IDIOPATHIC ARTHRITIS
31	1/1280		12	Female	2+	Ro-52 3+	AUTOIMMUNE HEPATITIS
32	1/1280	1/320 AC-1	13	Female	2+		JUVENIL IDIOPATHIC ARTHRITIS
33	1/1280	1/640 AC-1	13	Female	3+		FAMILIAL MEDITERRANEAN FEVER
34	1/1280		17	Female	3+		AUTOIMMUNE HEPATITIS
35	1/2560	1/1280 AC-4,5	4	Female	3+		FAMILIAL MEDITERRANEAN FEVER
36	1/2560	1/2560 AC-4,5	14	Female	3+	DFS 3+	JUVENIL IDIOPATHIC ARTHRITIS
37	1/2560		16	Female	1+		NON-RHEUMATIC ARTHRALGIA
38	1/2560		14	Female	3+		SYSTEMIC SCLEROSIS
39	1/2560		11	Female	3+		SYSTEMIC SCLEROSIS
40	1/5120	1/2560 AC-4,5	9	Male	3+		SYSTEMIC SCLEROSIS
41	1/5120	1/5120 AC-4,5	17	Female	3+		CELIAC DISEASE
42	1/5120	1/320 AC-1	16	Female	3+	Ro-52 2+	BEHCET DISEASE
43	NEGATIVE	1/160 AC-4,5	12	Female	1+		NON-RHEUMATIC ARTHRALGIA
44	NEGATIVE	1/160 AC-4,5	16	Male	1+		PRIMARY BILIARY CIRRHOSIS
45	NEGATIVE	1/320 AC-1	8	Male	1+		JUVENIL IDIOPATHIC ARTHRITIS
46	NEGATIVE	1/320 AC-6	15	Female	2+		NON-RHEUMATIC ARTHRALGIA
47	NEGATIVE	1/640 AC-4,5	13	Female	1+		NON-RHEUMATIC ARTHRALGIA
48	NEGATIVE	1/640 AC-4,5	13	Female	2+		SYSTEMIC SCLEROSIS
49	NEGATIVE	1/5120 AC-4,5	12	Female	2+	dsDNA 1+	BEHCET DISEASE
50	NEGATIVE	1/320 AC-2	16	Female	2+	DFS 3+	NON-RHEUMATIC ARTHRALGIA
51	NEGATIVE		11	Male	1+		NON-RHEUMATIC ARTHRALGIA
52	NEGATIVE	1/320 AC-1	17	Female	1+		NON-RHEUMATIC ARTHRALGIA
53	NEGATIVE		10	Male	1+		NON-RHEUMATIC ARTHRALGIA
54	NEGATIVE	1/80 AC-4,5	18	Male	1+		NON-RHEUMATIC ARTHRALGIA
55	NEGATIVE	1/1280 AC-1	7	Female	1+		NON-RHEUMATIC ARTHRALGIA
56	NEGATIVE		4	Female	1+		NON-RHEUMATIC ARTHRALGIA
57	NEGATIVE	1/640 AC-1	10	Female	2+		NON-RHEUMATIC ARTHRALGIA
58	NEGATIVE	1/80 AC-4,5	17	Female	2+		NON-RHEUMATIC ARTHRALGIA
